# Predicting COVID-19 pandemic waves including vaccination data with deep learning

**DOI:** 10.3389/fpubh.2023.1279364

**Published:** 2023-12-15

**Authors:** Ahmed Begga, Òscar Garibo-i-Orts, Sergi de María-García, Francisco Escolano, Miguel A. Lozano, Nuria Oliver, J. Alberto Conejero

**Affiliations:** ^1^Instituto Universitario de Matemática Pura y Aplicada, Universitat Politécnica de València, València, Spain; ^2^Departamento de Ciencia de la Computación e I.A., Universidad de Alicante, Alicante, Spain; ^3^Ellis Alicante, Alicante, Spain

**Keywords:** SARS-CoV-2, COVID-19, vaccination, computational epidemiology, data science for public health, recurrent neural networks, non-pharmaceutical interventions

## Abstract

**Introduction:**

During the recent COVID-19 pandemics, many models were developed to predict the number of new infections. After almost a year, models had also the challenge to include information about the waning effect of vaccines and by infection, and also how this effect start to disappear.

**Methods:**

We present a deep learning-based approach to predict the number of daily COVID-19 cases in 30 countries, considering the non-pharmaceutical interventions (NPIs) applied in those countries and including vaccination data of the most used vaccines.

**Results:**

We empirically validate the proposed approach for 4 months between January and April 2021, once vaccination was available and applied to the population and the COVID-19 variants were closer to the one considered for developing the vaccines. With the predictions of new cases, we can prescribe NPIs plans that present the best trade-off between the expected number of COVID-19 cases and the social and economic cost of applying such interventions.

**Discussion:**

Whereas, mathematical models which include the effect of vaccines in the spread of the SARS-COV-2 pandemic are available, to the best of our knowledge we are the first to propose a data driven method based on recurrent neural networks that considers the waning effect of the immunization acquired either by vaccine administration or by recovering from the illness. This work contributes with an accurate, scalable, data-driven approach to modeling the pandemic curves of cases when vaccination data is available.

## 1 Introduction

The COVID-19 pandemic was the first pandemic for which data related to the number of infections, deaths, hospitalizations, and other relevant variables were captured and reported daily in over 100 countries in the world (1, 2). Data scientists across the globe, working with mathematicians and epidemiologists, developed computational models to predict the pandemic spread using a variety of approaches, including compartmental meta-population (e.g., SIR or SEIR) (3–6), statistical (7–10), agent-based (11–14), and deep learning-based (15–18) models. These models consider the impact of the applied non-pharmaceutical interventions (NPIs) and thus enable running simulations of what-if scenarios where different NPIs were to be applied.

The SARS-CoV-2 outbreak in Wuhan was made public on 31 December 2019. Its impact and spreading potential were early noticed (19), and the virus genome was sequenced at an early stage of the pandemic spread, showing its most remarkable features (20). The first vaccines were quickly developed due to a concerted effort by pharmaceutical companies, scientists, and governments. Clinical trials started in recorded time after the coronavirus pandemic was declared (21–24). This allowed the first vaccine doses to be available at the end of 2020 and the beginning of 2021 (25–27).

Estimating the immunity provided by the different vaccines before setting up vaccination plans was critical in preventing the spread of the infection and in estimating the reduction of the breakthrough infection and other indirect effects. These estimations across different population groups led to the proposal of specific vaccination strategies (28). Another factor to consider is each vaccine's decrease in immunity over time (29, 30).

Several mathematical models that leverage such information have been proposed to forecast the evolution of the pandemic under different vaccination policies worldwide, such as (15, 31). Immunity can be estimated in terms of confidence intervals, but, as described later, the waning in immunity may be modeled through Weibull distributions (32).

However, we are not aware of any deep learning-based approach to predict the evolution of the COVID-19 pandemic while considering the impact of vaccination. In this study, we present a deep learning-based COVID-19 case predictor that includes vaccination data and thus extends the previous study by (17, 18).

We empirically test different implementations with data from the first quarter of 2021 when vaccines started to be available. At that time, the predominant variants of SARS-CoV-2 were Alpha, Beta, and Gamma, which were closer to the variant considered to develop the vaccines than the Delta variant.

This study is organized as follows: In Section 2, we present the notation and the core computational epidemiological models used by our predictor. The data sources used for this study are described in Section 3. Section 4 presents the deep learning-based architecture that we used to implement the different models to predict the number of daily COVID-19 cases. Section 5 summarizes our results, followed by our conclusion in Section 6.

## 2 Computational epidemiological model

### 2.1 Notation

We will use the following terms and notation as per (17). Given an arbitrary country denoted by *GEO*_*j*_, we assume that its population is constant and denoted by *P*^*j*^. Its daily number of new COVID-19 confirmed cases on the *n*−*th* day, starting from 1st September 2020, will be denoted by $X_{n}^{j}$. In our estimations, we will consider the smoothed averaged number of cases between the days *n*−*K*+1 and *n*, computed as $Z_{n}^{j}=\frac{1}{K}\overset{K-1}{\underset{i=0}{\sum }}X_{n-1}^{j}$, with *K* = 7, to smooth over 1 week.

Beyond the number of infected individuals on the *n*-th day at *GEO*_*j*_, we also consider $S_{n}^{j}$, the number of susceptible individuals who can be infected on the *n*-th day; $V_{n}^{j}$, the number of individuals protected by a vaccine on the *n*−*th* day; and $D_{n}^{j}$, number of retired (recovered or deceased) individuals in *GEO*_*j*_ on the *n*−*th* day. We compute the ratio of cases between 2 consecutive days as $C_{n}^{j}=Z_{n}^{j}/Z_{n-1}^{j}$, which shows the growth/decrease in the number of cases, and the rescaled ratio by the proportion of susceptible individuals, denoted by $R_{n}^{j}=C_{n}^{j}\frac{P^{j}}{S_{n}^{j}}$. This last quotient captures the effects of a finite population, as it depends on the proportion of susceptible individuals.

We denote the estimations provided by our models with a $\hat{\cdot }$ symbol, e.g., ${\hat{X}}_{n}^{j}$ denotes the estimated number of new COVID-19 cases on the *n*-the day in *GEO*_*j*_, and ${\hat{R}}_{n}^{j}$ the estimated scaled case ratio. Next, we present the two underlying computational epidemiological models in which our deep neural network models are based.

### 2.2 Compartmental SIR model

The classic compartmental metapopulation SIR model computes the number of Susceptible (S), Infected (Z), and Recovered (D) individuals as per the following differential equations:


(1) $$\begin{array}{lll} \frac{dS}{dt} & = & -\beta \frac{S}{P}Z+\sigma \left(D\right), \end{array}$$

(2) $$\begin{array}{lll} \frac{dZ}{dt} & = & \beta \frac{S}{P}Z-\mu Z. \end{array}$$

(3) $$\begin{array}{lll} \frac{dD}{dt} & = & \mu Z-\sigma \left(D\right) \end{array}$$

where β is the infection rate, μ is the recovery or removal rate, and σ(*D*) is a function of the retired individuals. This term is not usually included in basic SIR model formulations, but as the pandemic evolved, it is necessary to include it. The infection rate β, and thus $R_{n}^{j}$, depend on the transmissibility rates of the different variants circulating in GEO *j* at time *n* and on the applied non-pharmaceutical interventions (NPIs) at GEO *j*. During the period under consideration, there were several variants of concern (VOC) (Alpha, Beta, and Gamma) which changed to variants being monitored (VBM) in September 2021 due to the emergence and expansion of the Delta variant since June 2021 (33). We assume that the three VBM variants behave as a single one. As explained below, the effect of β and μ will be captured jointly in $R_{n}^{j}$, thus estimating them individually is not necessary.

### 2.3 Compartmental SIR model with vaccination (SVIR)

The previous SIR model can be extended to incorporate information regarding the level of vaccination in each GEO and the efficiency of the vaccines. It is given by the following equations:


(4) $$\begin{array}{lll} \frac{dS}{dt} & = & -\beta \frac{S}{P}Z+\sigma \left(D\right)-\alpha \left(P\right)+\gamma \left(V\right), \end{array}$$

(5) $$\begin{array}{lll} \frac{dV}{dt} & = & \alpha \left(P\right)-\gamma \left(V\right), \end{array}$$

(6) $$\begin{array}{lll} \frac{dZ}{dt} & = & \beta \frac{S}{P}Z-\mu Z, \end{array}$$

(7) $$\begin{array}{lll} \frac{dD}{dt} & = & \mu Z-\sigma \left(D\right). \end{array}$$

This model has two additional terms with respect to the previous one: α(*P*), which represents the daily vaccinated population, and γ(*V*), which is a function indicating the vaccinated population that becomes susceptible to the virus due to the waning effect of the vaccines.

From the discrete version of $\frac{dZ}{dt}$, either in 2 or 6, $Z_{n}^{j}$, the number of infected individuals on the *n*-th day in *GEO*_*j*_ is given as follows:


(8) $$\begin{array}{lll} Z_{n}^{j} & = & Z_{n-1}^{j}+\beta \frac{S_{n-1}^{j}}{P^{j}}Z_{n-1}^{j}-\mu Z_{n-1}^{j} \end{array}$$

(9) $$\begin{array}{ll} = & \left(1+\beta \frac{S_{n-1}^{j}}{P^{j}}-\mu \right)Z_{n-1}^{j}, \end{array}$$

where $S_{n-1}^{j}$ and $Z_{n-1}^{j}$ are the numbers of susceptible and infected individuals *GEO*_*j*_ on the day *n*−1, β is the infection rate, and μ as the recovery or removal rate, which yields the scaled case ratio, $R_{n}^{j}$ as in (16, 17):


(10) $$\begin{array}{l} R_{n}^{j}=\frac{Z_{n}^{j}}{Z_{n-1}^{j}}\frac{P^{j}}{S_{n}^{j}}=\frac{\left(1-\mu \right)P^{j}}{S_{n}^{j}}+\beta . \end{array}$$

Given that μ is constant in (10), the larger the infection rate β is, the larger the $R_{n}^{j}$ will be. If we predict $R_{n}^{j}$, we can estimate the number of COVID-19 cases for the *n*-th day in *GEO*_*j*_ as follows:


(11) $$\begin{array}{l} {\hat{X}}_{n}^{j}=\left({\hat{R}}_{n}^{j}\frac{S_{n-1}^{j}}{P^{j}}-1\right)KZ_{n-1}^{j}+X_{n-7}^{j}. \end{array}$$

It is worth mentioning that $Z_{n}^{j}$ is the resulting smoothed number of infected people on *GEO*_*j*_ over 7 days, from *n*−6 up to *n*. Moreover, $X_{n-7}^{j}$ is the real number of infected people on the day *n*−7 in *GEO*_*j*_.

While ${\hat{X}}_{n}^{j}$ is given by the same expression both in the SIR (1) and SVIR (4) models, the estimation of the number of susceptible individuals, $S_{n}^{j}$, is different due to the vaccination. In the case of the SVIR model, the total population *P*^*j*^ for *GEO*_*j*_ is given by $P^{j}=S_{n}^{j}+V_{n}^{j}+Z_{n}^{j}+D_{n}^{j}$, for any *n*∈ℕ, indicating that the total population on *GEO*_*j*_ is split on day *n* as the sum of the susceptible ($S_{n}^{j}$), vaccinated ($V_{n}^{j}$), infected ($Z_{n}^{j}$), and removed individuals ($D_{n}^{j}$), including both immunized and deceased individuals. Thus, discretizing $\frac{dS}{dt}$, the number of susceptible individuals on the *n*-th day in *GEO*_*j*_, denoted as $S_{n}^{j}$, can be obtained as follows:


(12) $$\begin{array}{l} S_{n}^{j}=S_{n-1}^{j}-Z_{n-1}^{j}-\alpha {\left(P\right)}_{n-1}^{j}+\sigma {\left(D\right)}_{n-1}^{j}+\gamma {\left(V\right)}_{n-1}^{j}, \end{array}$$

where $\alpha {\left(P\right)}_{n-1}^{j}$ represents the total number of vaccinated individuals on the day *n*−1, $\gamma {\left(V\right)}_{n-1}^{j}$, reflects the vaccinated individuals who have lost immunity on day *n*−1, and $\gamma {\left(D\right)}_{n-1}^{j}$ corresponds to the infected individuals who have lost immunity on day *n*−1.

The impact of the loss on immunity by part of the population is complex and hard to infer, as it depends on the types of vaccines delivered in each GEO, the distribution of variants with their respective infection rates, the number of doses administered, and the number of partial and fully vaccinated individuals (34). For instance, the Alpha variant was predominant with respect to the Primal variant between January 2021 and June 2021, when the Delta variant became a variant of concern (33). In our experiments we assume that:

(1) All circulating SARS-CoV-2 variants are a unique variant during the entire period of study; and
(2) All vaccines impact individuals equally, independently of their age, gender, or ethnicity, given that such information is not available in the compartmental metapopulation models.

### 2.4 Decay over time in the vaccine's immunity to SARS-CoV-2 infections

The decay of the vaccine's immunity against a SARS-CoV-2 infection may be fitted using a Weibull or a lognormal model. Both of them estimate a similar average protection, but the Weibull model provides a slightly better fit over time (32). The waning effect of the vaccine's immunity on day *n* is modeled by the means of a Weibull distribution of parameters *k* and ρ for the following eight vaccines:

1. ChAdOx1 (Oxford/Astrazeneca, OA)
2. Ad5-nCoV Convidecia (Cansino, CA)
3. mRNA-1273 (Moderna Biotech, MO)
4. BBIBP-CorV (Sinopharm, SP)
5. CoronaVac (Sinovac, SV)
6. Sputnik V/Gam-COVID-Vac (Gamaleya, GA)
7. Ad26.COV2.S (Janssen, JA)
8. BNT162b2 (Pfizer/BioNTech, PB)

We denote by


(13) $$\begin{array}{l} F\left(n,\lambda_{i},k_{i}\right)=e^{-{\left(n/\lambda_{i}\right)}^{k_{i}}} \end{array}$$

the complement of the Weibull distribution that models the waning effect on day *n* of each of the eight vaccines listed above. These models are known as accelerated failure time models and are frequently used in survival analyses. We use the same fitting parameters λ_*i*_ and *k*_*i*_ as those reported in the study mentioned in the reference (32, Table 4). As shown in the Table 1, the parameter estimates are available for individuals who are vaccinated with either a complete or incomplete dose and for actively infected individuals.

**Table 1:** Fitted parameters for the Weibull distribution (λ, *k*), for complete and incomplete doses from (32).

**Vaccine**	**Complete dose**		**Incomplete dose**	
	**λ_*p*_**	***k*_*p*_**	**λ_*f*_**	***k*_*f*_**
1 (OA)	205.6	2.9	65.6	1.3
2 (CA)	166.0	2.0	63.5	1.15
3 (MO)	217.0	3.6	83.5	1.15
4 (SP)	191.0	2.7	73.2	1.15
5 (SV)	184.9	2.5	70.1	1.2
6 (GA)	206.2	2.9	77.5	1.2
7 (JA)	178.6	3.0	—	—
8 (PB)	235.3	2.7	92.0	1.1

**Figure 1:**
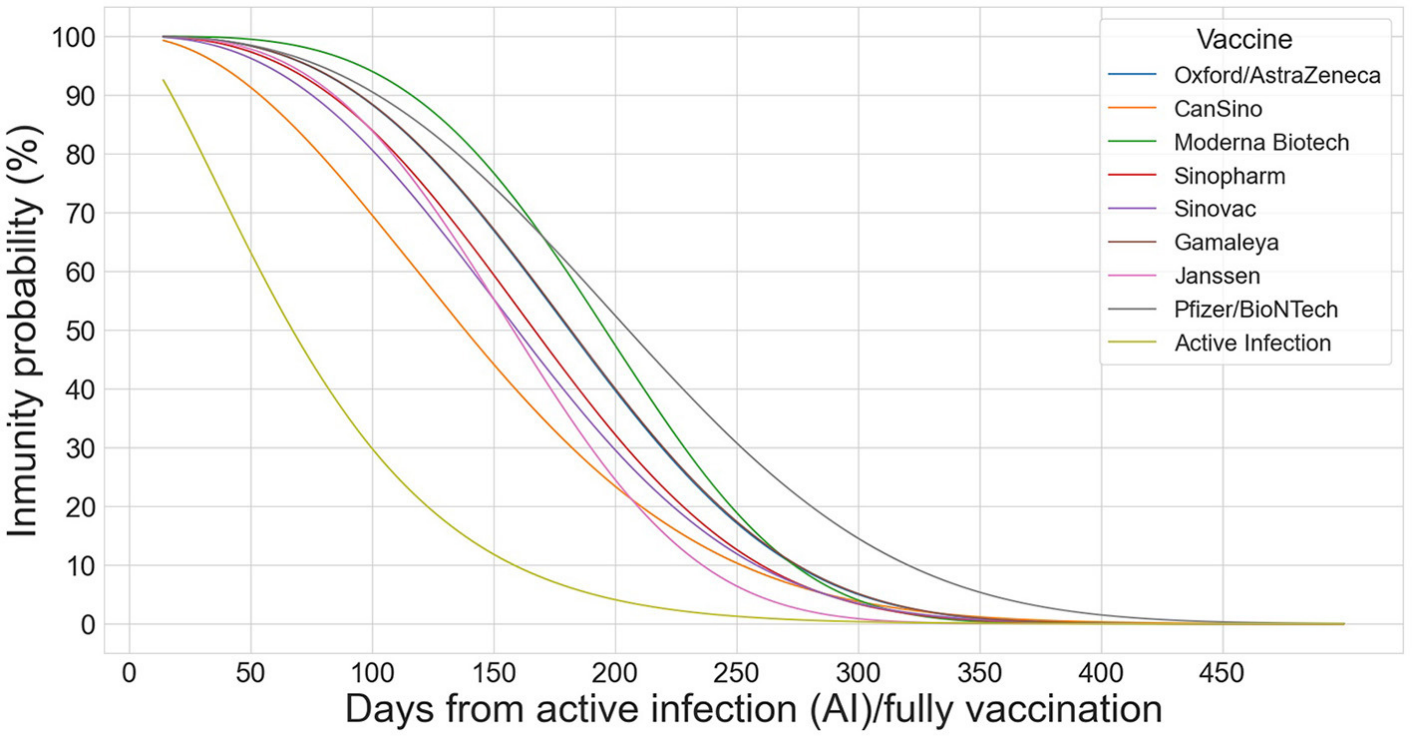
Weibull distributions to model the decay effect of the 8 vaccines (OA, CA, MO, SP, SV, GA, JA, and PB) on infected and fully vaccinated individuals.

Figure 1 shows the Weibull functions that model the probability of immunity for infected and fully vaccinated individuals and for each of the eight vaccines.

In Equation (13) and in the rest of the formulas, the index *i* = 0 represents the already infected population; *i*∈[1, 8] denotes each one of the eight vaccines, following the order in which they are listed above. We assume that: (1) protection starts on the 14th day after the last –complete or partial– dose; and (2) individuals can get reinfected after *d*_0_ = 14 days. Given these assumptions, the number of infected individuals who become susceptible again on *GEO*_*j*_ and on day *n* is given as follows:


(14) $$\begin{array}{l} \sigma {\left(D\right)}_{n}^{j}=\left(1-F\left(d_{0},\lambda_{0},k_{0}\right)\right)Z_{n-14} \end{array}$$

(15) $$\begin{array}{l} +\overset{n-d_{0}}{\underset{l=1}{\sum }}\left(F\left(d_{0}-1+l,\lambda_{0},k_{0}\right)-F\left(d_{0}+l,\lambda_{0},k_{0}\right)\right)Z_{n-l-14}, \end{array}$$

for *n*≥*d*_0_+1, where λ_0_ = 87.3 and *k*_0_ = 1.4 as per (32). The number of vaccinated individuals that become susceptible after waning immunity is computed as follows:


(16) $$\begin{array}{l} \gamma {\left(V\right)}_{n}^{j}=\underset{v=p,f}{\sum }\overset{8}{\underset{i=1}{\sum }}\left(1-F\left(d_{0},\lambda_{i,v},k_{i,v}\right)\right)V_{n-14}^{i}+ \end{array}$$

(17) $$\begin{array}{l} \underset{v=p,f}{\sum }\overset{8}{\underset{i=1}{\sum }}\overset{n-d_{1}}{\underset{l=1}{\sum }}\left(F\left(d_{0}-1+l,\lambda_{i,v},k_{i}\right)-F\left(d_{0}+l,\lambda_{i,v},k_{i,v}\right)\right)V_{n-l-14}^{i}, \end{array}$$

for *n*≥*n*_0_+*d*_0_ = 363, where $V_{s}^{i}$ is the number of individuals that were vaccinated on day *s* with vaccine *i*; *v* indicates whether individuals are partially (p) or fully vaccinated (f); and *n*_0_ corresponds to 14 December 2020 (349th day of the year) plus *d*_0_ days of latency until individuals may get infected again when the vaccination started worldwide.

## 3 Data sources

The number of infected and vaccinated individuals and the non-pharmaceutical interventions (NPIs) applied in each GEO of interest were retrieved from the Oxford COVID-19 Government Response Tracker (OxCGRT) (35). If a country has a negative number of cases in 1 day, we replace this number with 0. The input to the prediction model is the smoothed number of cases obtained by computing their average over 7 days.

Table 2 shows the NPIs considered in this study. They are categorical variables that indicate the level of intensity of applying each NPI: the higher the level, the more restrictive the applied measure is. Detailed information about these levels can be found in the codebook of the OxCGRT (35) and in the Supplementary material of (17).

**Table 2:** NPIs considered in this study and their possible activation values.

**NPI name**	**Values**
C1. School closing	[0, 1, 2, 3]
C2. Workplace closing	[0, 1, 2, 3]
C3. Cancelation of public events	[0, 1, 2]
C4. Restrictions on gatherings	[0, 1, 2, 3]
C5. Close public transport	[0, 1, 2]
C6. Stay at home requirements	[0, 1, 2, 3]
C7. Internal movement restrict.	[0, 1, 2]
C8. Intl. travel controls	[0, 1, 2, 3]
H1. Public info. campaigns	[0, 1, 2]
H2. Testing policy	[0, 1, 2, 3]
H3. Contact tracing	[0, 1, 2]
H6. Facial coverings	[0, 1, 2, 3, 4]
H7. Vaccination policy	[0, 1, 2, 3, 4, 5]

See the codebook of the OxCGRT (35) for the NPI-level description associated with each categorical value.

One of these NPIs (H7) describes the population groups that are covered by vaccination with the following levels: (0) vaccines are not available; (1–3) vaccines are available to one or more of the following groups (indicating the number of them): key workers, clinically vulnerable groups, and older individuals; (4) vaccines are available for broader groups; and (5) vaccines are universally available. The complete description of each NPI can be found at the study mentioned in the reference (36). All the predictor models described in this study consider all confinement (C1 to C8) and some public health interventions (H1 to H3 and H6). The vaccination NPI (H7) may be used to incorporate vaccination into an SIR model or complement an SVIR model, as explained below.

The number of administered vaccine doses per GEO and day is obtained from the OxCGRT dataset. However, this information is not provided per vaccine type. We obtained the vaccine specific details from the study mentioned in the reference (2, 37) but only for the following GEOs: Argentina, Austria, Belgium, Bulgaria, Canada, Croatia, Cyprus, Czech Republic, Denmark, Ecuador, Estonia, Finland, France, Germany, Hungary, Ireland, Italy, Latvia, Lithuania, Luxembourg, the Netherlands, Norway, Poland, Portugal, Slovak Republic, Slovenia, Spain, Sweden, Switzerland, and United States. In the following, we refer to these countries as GEOs, being *GEO*_*j*_ the j-th country in this set. Once we have defined the underlying computational epidemiological models and described the data sources, the next sections present the different implementations of the deep learning-based predictor of daily COVID-19 cases and their evaluation with real data.

## 4 Predictors of COVID-19 cases with vaccination

### 4.1 Basic architecture

**Figure 2:**
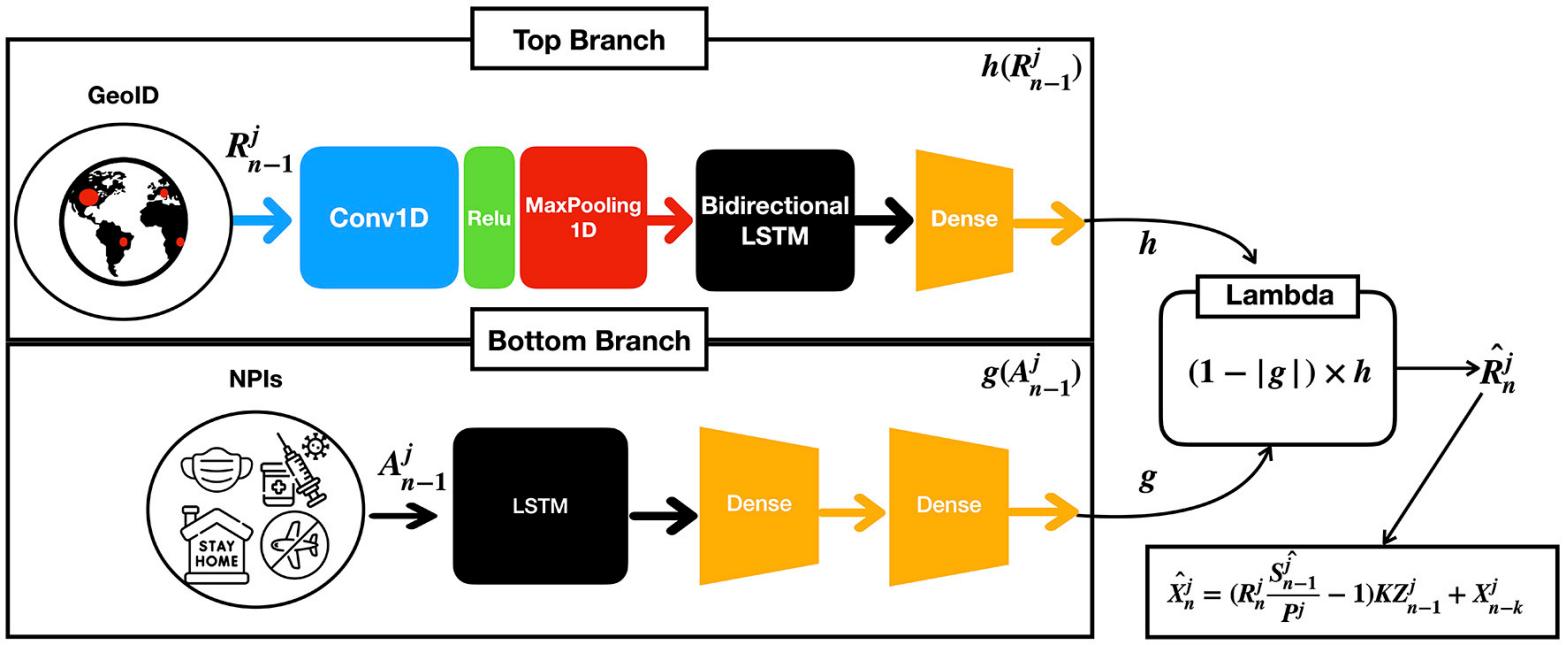
Given the previous contexts $R_{n-1}^{j}$ and actions $A_{n-1}^{j}$ on GEO *j* up to the *n*−1-th day, the model computes an estimated ${\hat{R}}_{n}^{j}$ which is the infection rate at *n*-th day for GEO j as a result of combining both branches with a lambda function.

We base our predictor architecture in the architecture presented in the study by (17). It consists of two parallel branches of bidirectional Long Short Term Memory layers (LSTM) (38), as shown in Figure 2: one (top branch) to predict $R_{n}^{j}$, i.e., the COVID-19 infection rate in *GEO*_*j*_ on day *n* (context), and the other (bottom branch) to model the effect of the applied NPIs (actions), $A_{n}^{j}$. Each LSTM provides separate predictions from the context, denoted by *h* and actions, denoted by *h*, combined using a lambda layer to yield an estimated ${\hat{R}}_{n}^{j}$. From ${\hat{R}}_{n}^{j}$, the number of daily cases is computed as per Equation (11). While we obtain a model for all the GEOs, for conducting predictions on each GEO, we use its own context and action data. The model is implemented in TensorFlow and Keras, running in a computer with an RTX 3090 GPU with 24 GB of RAM.

The architectural details of each of the branches are as follows:

1. The context branch (top) consists of a one dimensional convolutional layer with the ReLu activation function, followed by a maxpool layer with pool size equal to two, and a bidirectional LSTM followed by a dense layer. The convolutional layer has 64 filters with kernel of size 8, and the bidirectional LSTM with 32 units encodes the input sequence into states of 32 dimensions, which are then provided to the dense layer for prediction. This architecture empirically generalized well for many GEOs, achieving good performance in both short- and long-term predictions (17). The outcome of this layer is denoted by a function *h* in terms of the ratios of cases $R_{n}^{j}$.
2. The action branch (bottom) consists of an LSTM followed by two dense layers to capture non-linearities. We use a sigmoid activation function to ensure the output is in the [0, 1] range. The outcome of this layer is denoted by a function *g*(*A*) in terms of the NPi's $A_{n}^{j}$ applied in the *GEO* *j*. Moreover, we constrain *g(A)* to satisfy the following condition: if the difference between two sets of actions *A* and *A*′ is greater than or equal to 1, (1−*g*(*A*)) must be lower or equal to (1−*g*(*A*′)).
3. Finally, a lambda layer combines the outcomes of the context and action branches and provides the predictions of $\hat{R_{n}^{j}}$ that permits estimating future cases.

### 4.2 Enhanced models with vaccination

We introduce two key modifications with respect to previously described basic model. First, the rapid expansion of the Alpha/Delta/Omicron variants enables learning a context model for all GEOs simultaneously instead of clusters of countries. Second, instead of a traditional SIR model, we include vaccination information in two ways: (1) through an NPI (H7) as an action in the action branch or (2) with an SVIR model that considers the effects of vaccination. The hypothesis is that the SVIR model would yield more accurate predictions once vaccinations are widespread, as it considers the protective effect of vaccination. Nevertheless, as time goes by, the probability of reinfection increases, it is necessary to include waning immunity in the models.

We compare eight different predictors. First, we use the baseline model (Baseline 1) introduced in the study mentioned in the reference (16) and served as a baseline for the XPRIZE Pandemic Response Challenge. We also benchmark our proposed models against a second baseline model (Baseline2), the predictor presented in the study mentioned in the reference (17) but without performing any clustering of GEOs as we only consider the 30 GEOs, where vaccination data were available as opposed to 198 GEOs. With such a limited number of GEOs, a clustering process is unsuitable. In neither of these predictors, there is no reintroduction of infected individuals who have lost immunity.

In addition, we consider six predictors to test the different implementations of vaccination data. All the predictors consider a waning immunity of infected individuals. These are the models under consideration, according to the nomenclature used in Table 3:

1. *SIR w/o H7 w/o VacW*: SIR model that reintroduces infected individuals that lost immunity but that neither considers NPI H7 nor the waning in the vaccines' immunity.
2. *SIR H7 w/o VacW*: SIR model that reintroduces infected individuals that lost immunity and considers NPI H7 but does not consider the waning in the vaccines' immunity.
3. *SVIR w/o H7 w/o VacW*: SVIR model that reintroduces infected individuals that lost immunity but that neither considers NPI H7 nor the waning in the vaccines' immunity.
4. *SVIR w/o H7 & VacW*: SVIR model that reintroduces infected individuals that lost immunity considers the waning in the vaccines' immunity but does not include NPI H7.
5. *SVIR H7 w/o VacW*: SVIR model that reintroduces infected individuals that lost immunity and considers NPI H7 but does not consider the waning in the vaccines' immunity.
6. *SVIR H7 & VacW*: SVIR model that reintroduces infected individuals that lost immunity and considers both NPI H7 and the waning in the vaccines' immunity.

**Table 3:** Accuracy of the predictors expressed in terms of MAE and Mean Rank (M. Rank) from 1st January 2021 to 30th April 2021.

**Predictor**	**SIR**		**SVIR**	
	**MAE**	**M. Rank**	**MAE**	**M. Rank**
Baseline 1	24.87	4.10	—	—
Baseline 2	24.69	3.88	—	—
w/o H7 w/o VacW	14.56	3.47	12.45	2.80
w/o H7 & VacW	—	—	11.21	2.77
H7 w/o VacW	13.84	3.71	11.59	2.32
H7 & VacW	—	—	**10.98**	**1.86**

Bold values indicate the best results in terms of MAE and Mean Rank.

Notably, the SIR model only allows to include vaccination by adding NPI H7. In our experiments, we compare these predictors with real data in the 30 GEOs of study.

The input to the models consists of data from previous confirmed cases and the NPIs implemented in each GEO. The NPIs are represented as a vector of categorical values that indicate the strength of each of the interventions, as previously explained.

## 5 Results

In this section, we first present the results of testing the previously described models to predict the number of COVID-19 cases globally between January and April 2021. We train the predictor with data retrieved from OxCGRT data set to predict the daily COVID-19 cases for the aforementioned list of GEOs between 1st September 2020 and 30th April 2021. All models were trained starting in 1st September 2020 until the day before the first prediction day. The models have a cumulative error since the prediction for the first day is used to make the prediction for the second one. In our experiments, we observed that for prediction periods longer than a fortnight, the error in the predictions started to increase significantly. Thus, we trained a new predictor every 15 days in the testing period and tested it to predict the number of COVID-19 cases in the next 14 days. After summarizing, to predict the number of newly infected individuals on day *d*_0_, the models are trained with data up to *d*_0_−8. We run five simulations to predict the number of new infections for *d*_0_−7 to *d*_0_−1 days. We select the model with the lowest mean absolute error (MAE) and use it to predict the number of COVID-19 cases for the period *d*_0_ to *d*_0_+13. To prevent overfitting, we use a validation data set at each epoch at training and an early stopping callback such that when the validation MAE stops decreasing, the training process is also stopped.

Table 3 shows the MAE and Mean Rank of all the models, including the baselines ones. Notably, the MAE is normalized by 100,000 inhabitants to enable a fair comparison across GEOs independently of the population size. To compute the Mean Rank, the models are ranked on each GEO and period, assigning 1 to the best-performing model and a 7 to the worst-performing model. The mean of all ranks on all GEOS is computed to obtain each predictor's Mean Rank.

**Figure 3:**
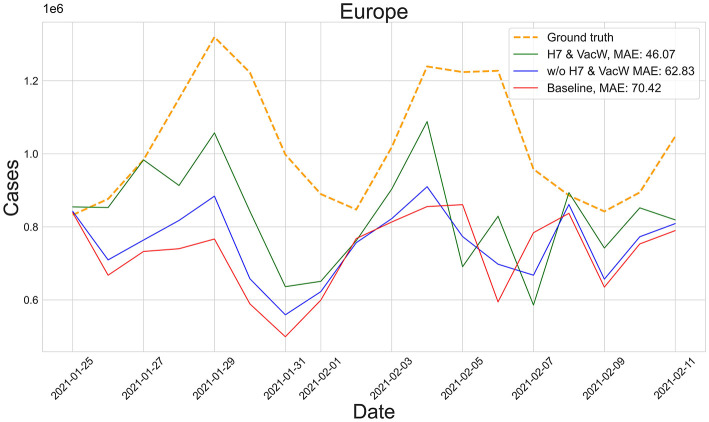
Predictions from 25th January to 11th February of the number of COVID-19 cases vs. the ground truth (yellow dashed line) for Europe with MAE per 100,000 inhabitants.

Figure 3 shows the predictions of the two best-performing predictors (H7 & VacW SVIR and w/o H7 & VacW SVIR) compared with the ground truth (yellow dashed line) and the baseline 2 model (red line), between mid-January and mid-February 2021, immediately after the vaccinations started to have an impact on the spread of COVID-19. Let us note how the inclusion of H7 improves the estimation.

**Figure 4:**
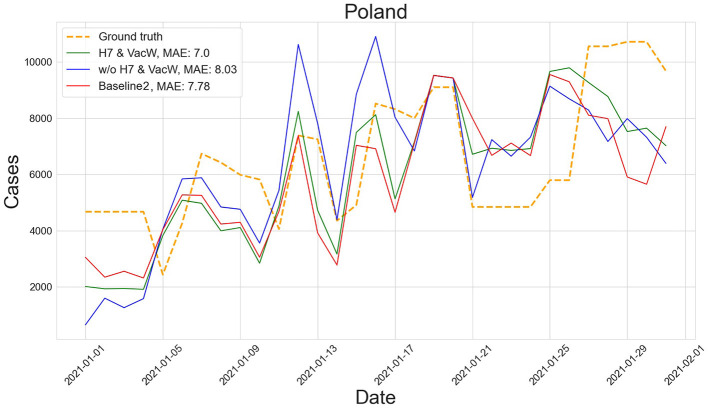
Prediction of the number of COVID-19 cases vs. the ground truth for Poland with MAE per 100,000 inhabitants in January 2021.

**Figure 5:**
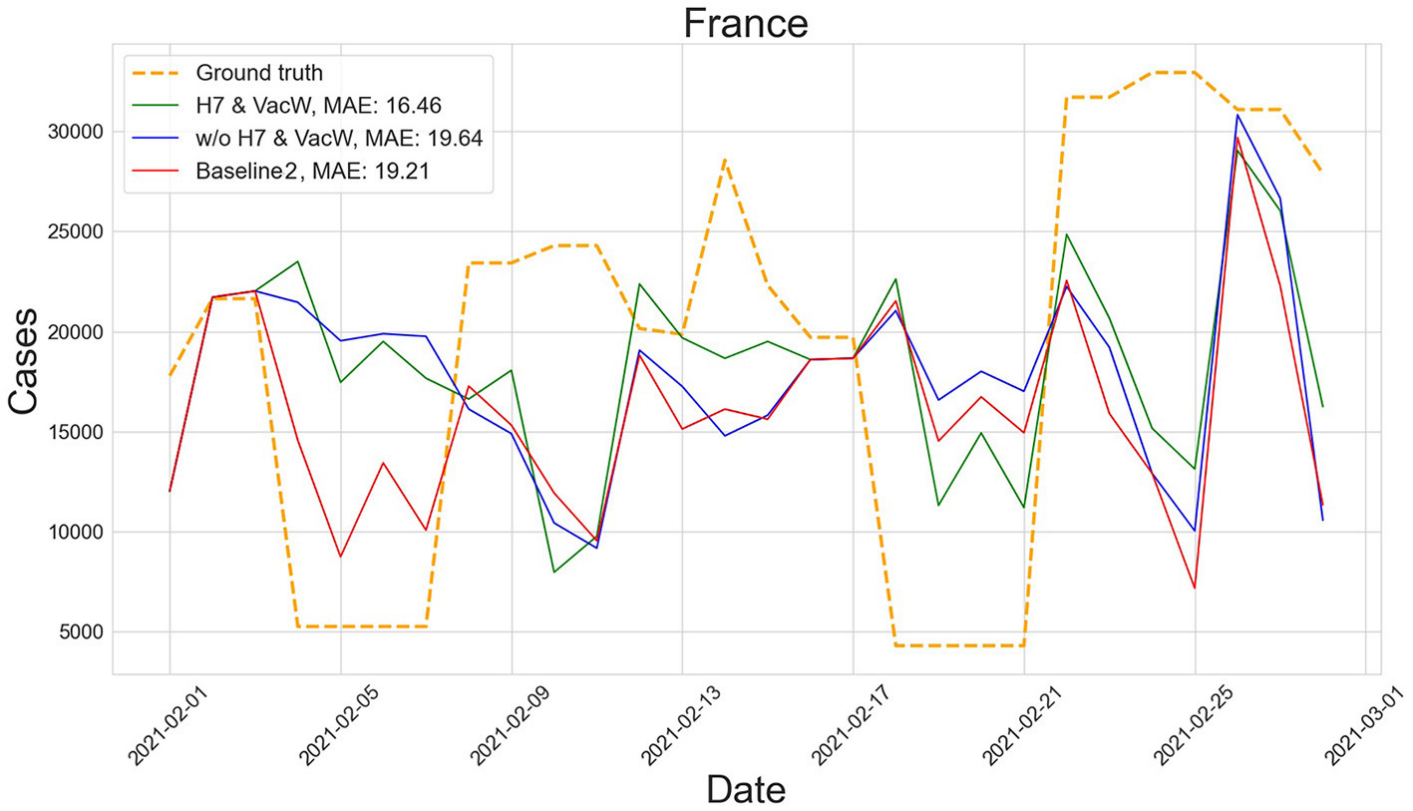
Predictions of the number of COVID-19 cases vs. the ground truth for France with MAE per 100,000 inhabitants in February 2021.

**Figure 6:**
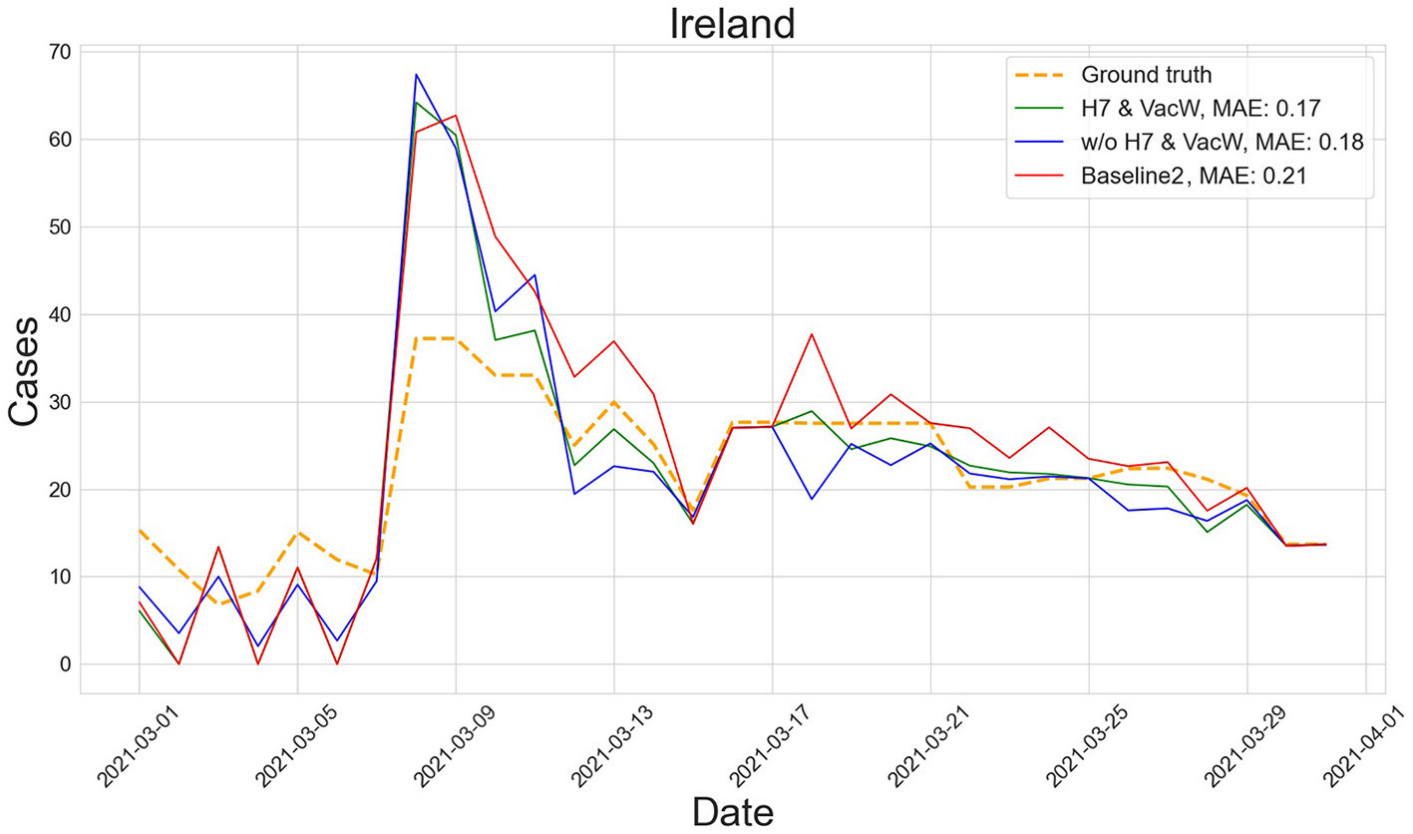
Predictions of the number of COVID-19 cases vs. the ground truth for Ireland with MAE per 100,000 inhabitants in March 2021.

**Figure 7:**
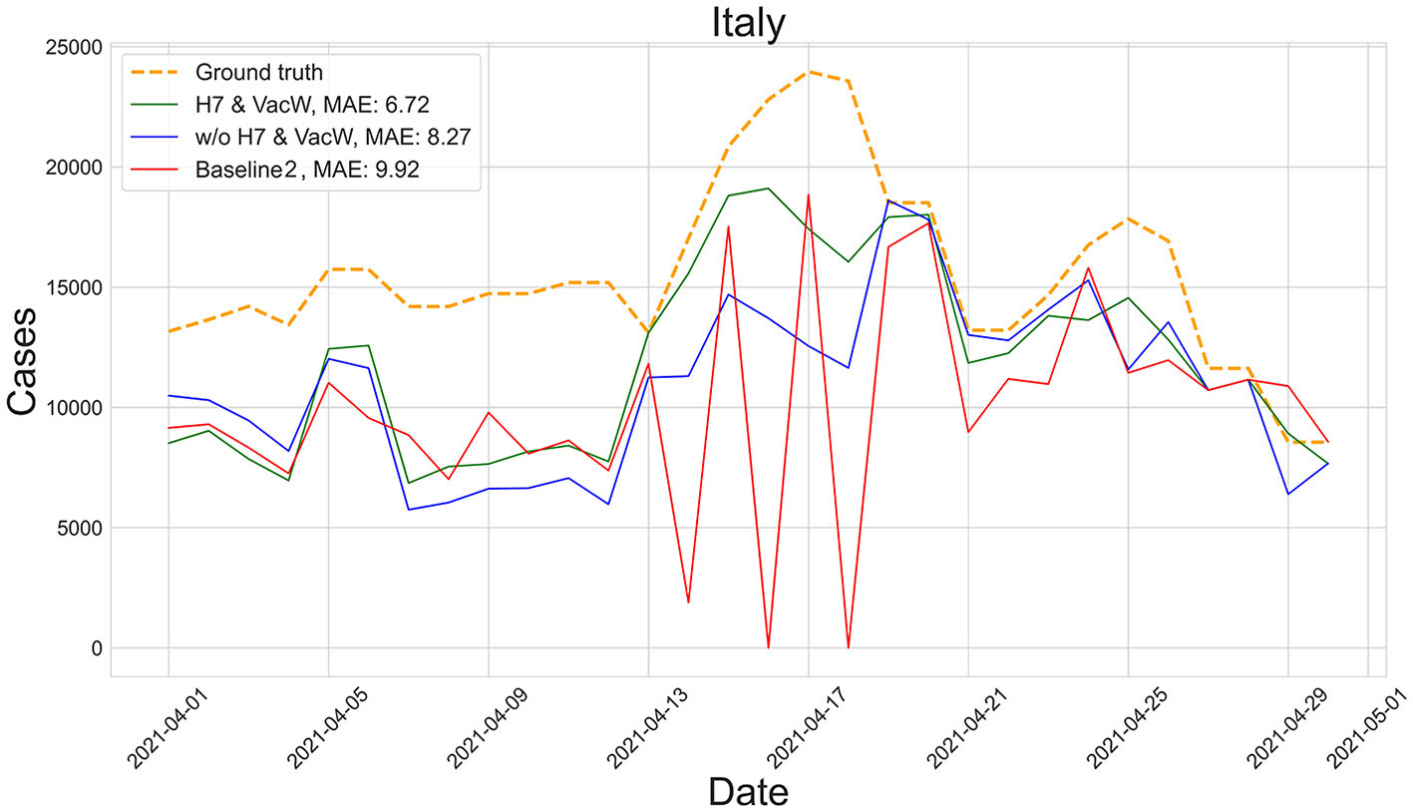
Predictions of the number of COVID-19 cases vs. the ground truth for Italy with MAE per 100,000 inhabitants in April 2021.

Figures 4–7 show the predictions of the two best-performing predictors on data between January and March of 2021 on several European countries with very different dynamics in the evolution of their number of COVID-19 cases: Poland during January 2021, when cases were increasing (Figure 4); France during February 2021, when cases were stabilized (Figure 5); Ireland during March 2021, when cases tended to decrease or stabilize (Figure 5); and Italy during April 2021, when there were two peaks of infections (Figure 7). Let us note how the H7& VacW SVIR predictor is able to correctly capture the trends in the pandemic curves even with such diversity of situations of the pandemic.

## 6 Conclusion

In this study, we have presented a deep learning-based predictor of COVID-19 cases in 30 countries that considers both the daily Non-Pharmaceutical Interventions applied in each country and vaccination data.

It is worth mentioning that despite the abundance of data, it is complex to consider information regarding age groups, doses administered of each vaccine, and the coexistence of different strains with different transmissibility rates, which were different from the primal strain used for designing the vaccines. In addition, the most efficient vaccines were the mRNA-based vaccines, which were the first ones to be designed and massively applied with this technology, and the duration of their effects on individuals from different regions is still under study, which may lead to potential biases (39).

Despite these difficulties and limitations, the proposed approach effectively considers vaccination information in a machine learning-based model that can be applied to different countries to predict the number of COVID-19 cases. Our models have shown a competitive performance over a long time period between January and April of 2021, when the vaccination campaigns started in many countries. Our study illustrates the value of having access to high-quality systematic data during a pandemic to enable evidence-driven decision-making.

All code and files used in this study are available at https://github.com/AhmedBegggaUA/frontiers_in_public_health.

## Data availability statement

The datasets presented in this study can be found in online repositories. The names of the repository/repositories and accession number(s) can be found below: https://github.com/AhmedBegggaUA/frontiers_in_public_health.

## Author contributions

AB: Conceptualization, Writing – original draft, Writing – review & editing, Data curation, Formal analysis, Methodology, Software, Validation, Visualization. ÒG-i-O: Data curation, Software, Writing – original draft, Writing – review & editing, Conceptualization, Formal analysis, Methodology, Validation, Visualization. SM-G: Data curation, Software, Formal analysis, Validation, Writing – review & editing. FE: Formal analysis, Funding acquisition, Software, Validation, Writing – review & editing. ML: Formal analysis, Funding acquisition, Software, Validation, Writing – review & editing. NO: Conceptualization, Formal analysis, Funding acquisition, Investigation, Software, Validation, Writing – original draft, Writing – review & editing. JC: Writing – original draft, Writing – review & editing, Conceptualization, Formal analysis, Funding acquisition, Investigation, Methodology, Software, Supervision, Validation.
